# Intestinal Transportations of Main Chemical Compositions of Polygoni Multiflori Radix in Caco-2 Cell Model

**DOI:** 10.1155/2014/483641

**Published:** 2014-02-12

**Authors:** Jie Yu, Na Li, Pei Lin, Yunfei Li, Xiaojian Mao, Getuzhaori Bao, Wen Gu, Ronghua Zhao

**Affiliations:** Yunnan University of Traditional Chinese Medicine, Kunming, Yunnan 650500, China

## Abstract

*Context*. Polygoni Multiflori Radix (PMR) is originated from the root of *Polygonum multiflorum *Thunb. and used in oriental countries for centuries. However, little researches pay close attention to the absorption of its major constituents. *Objective*. Transepithelial transport of TSG, RL, PL, and four anthraquinones is carried out. *Materials and Methods*. Caco-2 cell monolayer, which represented a well-established model for the study of intestinal transport of nutrients and xenobiotics, was used in this paper. *Results*. The apparent permeability coefficients (*P*_app_) in the Caco-2 cell monolayers were TSG (2.372 × 10^−9^) < EG (2.391 × 10^−9^) < EN (2.483 × 10^−9^) < PL (4.917 × 10^−9^) < RN (1.707 × 10^−8^) < RL (1.778 × 10^−8^) < AE (1.952 × 10^−8^). Thus, RN, RL, and AE were considered partly absorbed, while other constituents were hardly absorbed. *Discussion and Conclusion*. Glycosides showed poor permeabilities than aglycones. In the meantime, TSG and EN gave out poor recovery rates in this assay, which indicated that TSG and EN may accumulate or metabolise in the Caco-2 cells. *In silico *prediction indicated that Gibbs energy (*r* = 0.751, *p* < 0.05) and heat of form (*r* = 0.701, *p* < 0.05) were strongly positively correlated with *P*_app_.

## 1. Introduction

**Figure 1:**
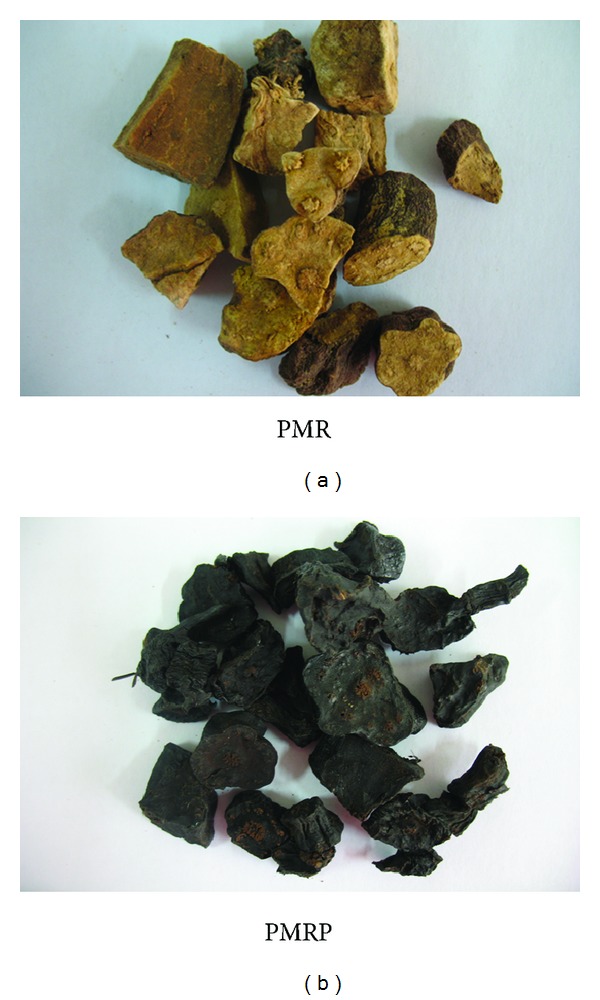
Photographs of Polygoni Multiflori Radix and its processed products. (a) PMR: *Polygonum Multiflorum* Radix. (b) PMRP: Polygoni Multiflori Radix Praeparata.

Polygoni Multiflori Radix ((PMR), heshouwu in Chinese) and Polygoni Multiflori Radix Praeparata ((PMRP), zhiheshouwu in Chinese) are originated from the root of *Polygonum multiflorum* Thunb. (Polygonaceae) and used in the treatment of nonalcoholic fatty liver disease (NAFLD) and hyperlipidemia in oriental countries for centuries (Figure 1).

Preliminary researches [1–4] indicate that Polygoni Multiflori Radix mainly contains stilbene glycosides (2,3,5,4′-tetrahydroxystilbene-2-*O*-*β*-D-glucoside (TSG), resveratrol (RL), polydatin (PL), and others) and anthraquinones (emodin (EN), rhein (RN), aloe-emodin (AE), emodin-8-*O*-*β*-D-glucopyranoside (EG), and others). In our previous research, TSG displayed the most important role in the total cholesterol (TC) lowering effect among all the chemical constituents of *Polygonum multiflorum*. The quality of PMR was evaluated by the contents of TSG and anthraquinones as regulated by the Pharmacopoeia of the People's Republic of China, 2010 edition [5].

However, little researches pay close attention to the absorption of these major constituents of PMR. In this research, transepithelial transport of TSG, RL, PL, and the four anthraquinones is carried out using human Caco-2 cell monolayer as a model system. Caco-2 cells, derived from a human colon adenocarcinoma, spontaneously differentiate after reaching confluence in culture, exhibiting several morphological and functional characteristics of mature enterocytes. Caco-2 cell monolayers represent a well-established model for the study of intestinal transport of nutrients and xenobiotics and are widely used in pharmacology and toxicology researches [6–10]. This research provided important predictive information regarding the oral bioavailability of TSG, RL, PL, EN, RN, AE, and EG.

## 2. Materials and Methods

### 2.1. Caco-2 Cell

Caco-2 cell (the human colon adenocarcinoma cell) was purchased from Kunming Institute of Zoology, Chinese Academy of Sciences, in June 2009.

### 2.2. Chemicals

**Figure 2:**
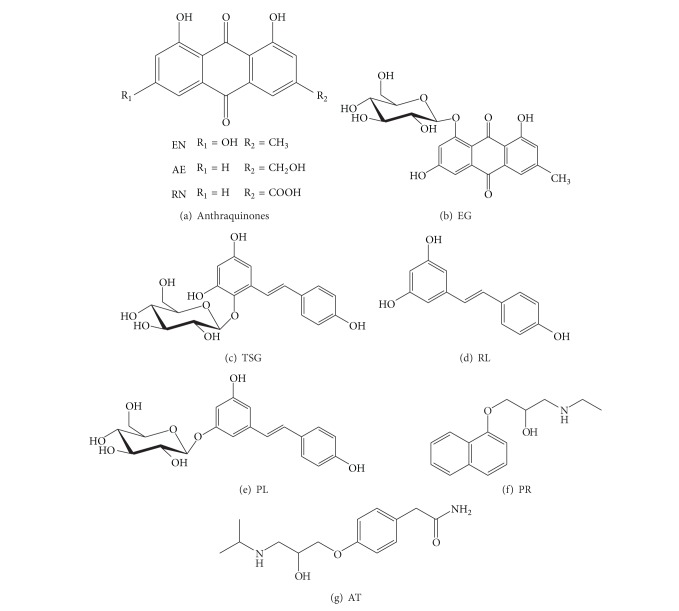
Structures of analytes in this research.

TSG, EN, RN, AE, RL, and PL were purchased from National Institute for the Control of Pharmaceutical and Biological Products, China. EG was purchased from the Sichuan Xianxin Biotech Co., Ltd., China. The purities of all the standards were not less than 98%. Propranolol (PR) was used as positive control substance of high permeability. Atenolol (AT) was used as positive control substance of poor permeability. Both PR and AT were purchased from Sigma. Structures of them were listed in Figure 2.

Dulbecco's Modified Eagle's Medium (DMEM) and fetal bovine serum (FBS) were obtained from Gibco Invitrogen Corporation. Phosphate buffer solution (PBS), N-2-hydroxyethylpiperazine-N′-2-ethanesulfonic acid (HEPES), L-glutamine, and pyruvic acid sodium salt were of chemical or analytical grade obtained from domestic company. Dimethyl sulfoxide (DMSO) was purchased from Sigma Chemical Co. (Deisenhofen, Germany).

### 2.3. Culture Conditions

Caco-2 cells were cultured in DMEM containing 1 g/L D-glucose, 2 g/L NaHCO_3_, 165 mg/L pyruvic acid sodium salt, and 150 mg/L L-Glutamine, supplemented with 10% FBS. Cultures were maintained in a humidified incubator with 5% carbon dioxide 95% air at 37°C. 0.25% of trypsin (Sigma, USA) (0.25%) was used to passage cells at 80–90% confluence and seeded at a density of about 1 × 10^5^ cells/mL on a 12-well Transwell (Corning Costar, Cambridge, MA, USA) insert filter (surface area = 1.13 cm^2^, pore size = 3 *μ*m). The cells were left to grow for 21 days to reach confluence and differentiation, so that Caco-2 cells were fully differentiated in this assay and used for further experiments. All cells used in this study were between passages 20 and 35.

### 2.4. Standardized Conditions

The Caco-2 cell system was validated by the transepithelial electrical resistance (TEER) assay (by EVOM2 from the World Precision Instrument Trading Co., Ltd.) and the alkaline phosphatase (ALP) activity difference between the two sides of the membrane. The TEER values >500 Ω/cm were required. The activities of ALP, as a specific brush border formation enzyme marker, in both AP and BL sides were estimated. The ALP_AP_/ALP_BL_ ratio was calculated.

### 2.5. Permeability Studies

The stock solutions of all tested compounds were achieved by dissolving them in dimethylsulfoxide (DMSO) at a concentration of 5 mmol/mL. Then they were further diluted with PBS to graded concentrations of 100 *μ*M and 200 *μ*M.

The following experiment was undertaken to measure the flux of the standards. Flux describes the movement of a substance across polarized Caco-2 monolayers either in absorptive (apical → basolateral, AP → BL) or secretary direction (basolateral → apical, BL → AP). Absorption assay was carried out in this research. The monolayers were washed twice with warm transport medium PBS within 30 min and then sequentially incubated once for 30 min at 37°C with PBS. Transport medium (0.5 mL) containing TSG, EN, RN, AE, RL, PL or EG was added to the AP side, while the receiving chamber contained the corresponding volume (1.5 mL) of transport medium. After shaking at 55 r/min for 1.5 h at 37°C in a shaking water bath, samples were collected (0.4 mL and 1.2 mL in 1 der constituents the AP and BL sides, resp.) from both sides of the Caco-2 cell monolayers and immediately frozen, lyophilized, and preserved below −20°C [11].

### 2.6. HPLC-DAD Analysis of Samples

To determine the concentration of corresponding constituents in both AP and BL sides, the lyophilized samples of AP and BL sides were dissolved in quantitative methanol by ultrasound for 20 min and then filtered by 0.45 *μ*m filtration membranes.

All experiments were performed with Dionex Ultimate 3000 HPLC system (Dionex Technologies, USA), which included a binary pump, an autosampler, a column oven, and a diode array detector plus on-line degasser. Data were analyzed with Chromeleon 6.8.

The separation of samples was achieved on Zorbax SB-C_18_ analytical column (4.6 × 250 mm, 5 *μ*m particle diameter, supplied by Agilent Technologies, USA).

Isocratic and gradient elution with mobile phase consisting of (A) 0.1% H_3_PO_4_ and (B) methanol was used. Methanol percentage and detection wavelength were listed in Table 1. The oven temperature was set at 30°C and the flow rate was set at 1.0 mL/min.

### 2.7. Standard Curve

The calibration curves for TSG, EN, RN, AE, RL, and PL were constructed by plotting concentration (*Y*, *μ*mol) versus peak area (*X*). The linear equation, linear range, and correlation coefficient were listed in Table 2.

### 2.8. Calculation of the Apparent Permeability Coefficient (*P*_app_) and Recovery Rate

The apparent permeability coefficient (*P*_app_) was calculated as follows:

(1) $$\begin{array}{c} P_{\text{app}}\left(\text{cm}/\text{s}\right)=\frac{dQ}{dt}\times \frac{1}{60}\times \frac{1}{A}\times \frac{1}{C_{0}}, \end{array}$$

where *P*_app_ was the apparent permeability coefficient in cm/s; *dQ*/*dt* was the rate of appearance of the test compound on the receiver side in *μ*mol/min; *C*_0_ was the initial concentration of test compound on the donor side in *μ*moL/L; and A was the surface area of the Transwell in cm^2^.

Meanwhile, the recovery rate of each compound was calculated as follows:

(2) Recovery  (%)=Total compound in donor and receiver at the end of the experiment (μM)Initial  compound  present  (μM) ×100%.

### 2.9. Quantitative Relationships between Physical-Chemical Parameter, Transepithelial Flux, and Recovery Rate

*In silico* prediction of physical, thermodynamic, and chemical parameters properties of analytes was made in this research. Basic physical and thermodynamic properties of these analytes, such as molecular weight, boiling point, Gibbs energy, and Henry's Law, were all predicted by ChemDraw Pro. Then we calculated the Log *P* (by ChemDraw Ultra 8.0), Clog  *P* (by ChemDraw Ultra 8.0, The OSIRIS Property Explorer, and The Bio-Lim., resp.), and solubility (by The OSIRIS Property Explorer) of these constituents.

Relationships between these physical, thermodynamic, and chemical parameters and *P*_app_ and recovery rate were assessed with Pearson's correlation coefficient. Results were classified into two significance levels using the *p* values of 0.05 and 0.01.

## 3. Results

### 3.1. The Integrity and Polarity of Caco-2 Cell Monolayer Membrane

**Figure 3:**
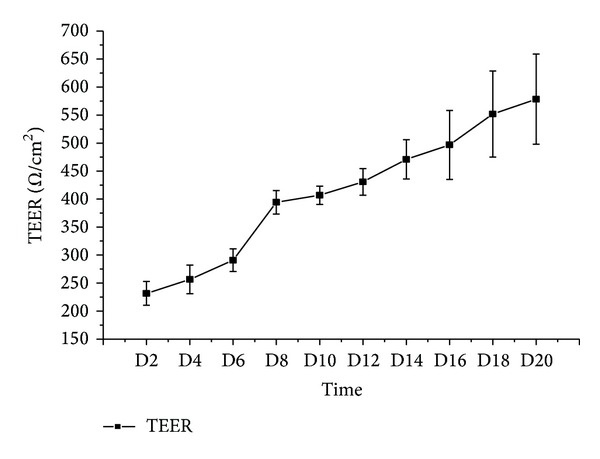
The value of transepithelial electrical resistance (TEER) (Mean ± SD, *n* ≥ 6, Ω/cm^2^).

The integrity of Caco-2 cell monolayer was evaluated by TEER value between AP side and BL side on alternate days through the 21-day culture period (Figure 3). The TEER value was rising continuously from the beginning to the 18th day of this research. The TEER value tended to be stable from the 18th day (551.7 ± 76.87) to the 20th day (578.4 ± 80.40), which indicated that a tight Caco-2 monolayer had been formed.

**Figure 4:**
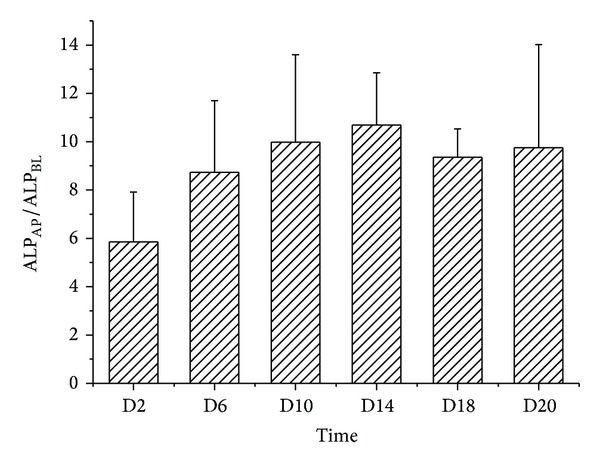
The alkaline phosphatase ratio (apical side to basolateral side, ALP_AP_/ALP_BL_) in this assay (Mean ± SD, *n* ≥ 6).

The ALP_AP_/ALP_BL_ ratio was increasing from the 2nd day to the 14th day and maintained in the maximum level from the 14th day to the 21st day (Figure 4). The activity of alkaline phosphatase in the AP side was almost ten times than that in BL side, which could further confirm the Caco-2 cells differentiation.

### 3.2. The *P*_app_ Values and Recovery Rate of TSG, EN, RN, AE, RL, PL, and EG through the Caco-2 Monolayer

**Figure 5:**
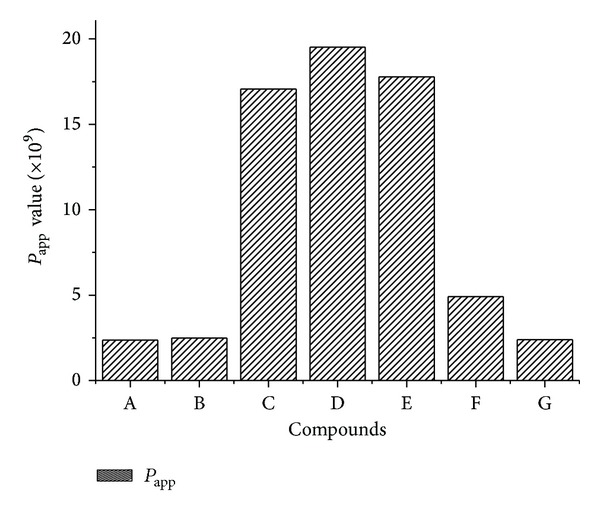
The apparent permeability coefficient (*P*_app_, cm/s) values of TSG, EN, RN, AE, RL, PL, and EG through the Caco-2 monolayer. A: 2,3,5,4′-tetrahydroxystilbene-2-*O*-*β*-D-glucoside (TSG), B: emodin (EN), C: rhein (RN), D: aloe-emodin (AE), E: resveratrol (RL), F: polydatin (PL), and G: emodin-8-*O*-*β*-D-glucopyranoside (EG).

The concentrations of analytes in AP and BL sides were analyzed after 90 mins incubation by HPLC-DAD equipment. The recovery rates and *P*_app_ values of seven compounds were listed in Table 3 and Figure 5.

RN, PR, and AT showed favorable recovery rates around 100%. AE, RL, PL, and EG exhibited middle extent (30~80%) recovery. EN and TSG gave out very poor recovery rate of only 14.48 ± 2.504% and 17.39 ± 1.600%. Usually, the poor recovery may indicate problems with poor solubility, binding of the compound to the plate, metabolism by the Caco-2 cells, or accumulation of the compound in the cell monolayer. We predict that large partitions of EN and TSG may accumulate or metabolise in the Caco-2 cells. However, further validation must be carried out to confirm this hypothesis.

Small *P*_app_ of TSG, EG, and PL (smaller than that of AT) reflected that they were hardly absorbed by human intestinal epithelial cells. This conformed with our consensus that glycosides were poorly absorbed than their aglycone due to the low fat solubility and low hydrophobicity. EN also showed small *P*_app_ probably due to its low recovery rate mentioned above. RN, RL, and AE showed middle *P*_app_ between atenolol (known human absorption of 50%) and propranolol (known human absorption of 90%) [12, 13]. Thus, RN, RL, and AE were considered partly absorbed. On the other hand, all the compounds were catalyzed to be passive-transported because the concentrations of each compound in the AP side were all higher than in the BL side.

### 3.3. Relationships between Physical-Chemical Properties Values and *P*_app_ and Recovery

Basic physical and thermodynamic properties of these analytes, such as molecular weight, boiling point, Gibbs energy, Henry's Law, Log *P*, Clog *P*, and Log *S*, were listed in Table 4.

Boiling point and melting point predicted the normal boiling and melting point for the structure, respectively. Critical temperature predicted the temperature (reported in K) above which the gas form of the structure cannot be liquefied, no matter the applied pressure. Critical pressure predicted the minimum pressure (reported as bars) that must be applied to liquefy the structure at the critical temperature. Critical volume predicted the volume occupied (reported in cm^3^/mol) at the compound's critical temperature and pressure. Heat of formation was the prediction of the heat of formation for the structure (reported in kJ/mol at 1 atm and 298.15 K). Gibbs energy was the prediction of the Gibbs free energy for the structure (reported in kJ/mol at 1 atm and 298.15 K).

The log *P* value of a compound, which is the logarithm of its partition coefficient between *n*-octanol and water (coctanol/water), was a well-established measure of the compound's hydrophilicity. Low hydrophilicities and therefore high log *P* values cause poor absorption or permeation. It has been shown for compounds to have a reasonable probability of being well absorbed their log *P* value must not be greater than 5.0 [14].

The aqueous solubility of a compound significantly affects its absorption and distribution characteristics. Typically, a low solubility goes along with a bad absorption and therefore the general aim is to avoid poorly soluble compounds. The Solubility Prediction (log *S*) was calculated by The OSIRIS Property Explorer in our research, in a unit stripped logarithm (base 10) of the solubility measured in mol/liter (shown in Table 3).

Pearson's correlation coefficients between these physical, thermodynamic, and chemical parameters and *P*_app_ and recovery were displayed in Table 5. Gibbs energy (*r* = 0.751, *p* < 0.05) and heat of form (*r* = 0.701, *p* < 0.05) were strongly positively correlated with *P*_app_. This was the first report to show that the permeability was strongly affected by the Gibbs energy and heat of form of a compound. Compounds with higher Gibbs energy and heat of form were considered to remain in higher energy state; therefore, these compounds had stronger tendency to complete the transmembrane movement. Moreover, boiling point, melting point, and critical temperature were all strongly positively correlated with *P*_app_ (*p* < 0.05).

## 4. Discussion and Conclusion

Previous studies [15, 16] reported that TSG has lipid regulation effect. Our previous researches [17, 18] also validated that the TSG possessed great TG reduction activity. Therefore, we could affirm that TSG is the main lipid regulation ingredient of PMR. However, in our previous studies, TSG content was significantly reduced after processed with black bean decoction (PMRP) according to the method recorded in the Pharmacopoeia of the People's Republic of China (2010 edition) [5].

In this research, TSG was estimated to be a poorly absorbed compound. The lower concentration of TSG in the processed PMRP together with its bad absorption all suggested that the dosage of PMRP should be increased in the treatment of hyperlipidaemia and NAFLD than the dosage of PMR. This coincided with the prescribed dosage listed in the Pharmacopoeia of the People's Republic of China (2010 edition), 3–6 g for PMR and 6–12 g for PMRP.

The human intestinal permeability of TSG, EN, RN, AE, RL, PL, and EG was evaluated using the Caco-2 cell monolayer model. In this research the permeabilities of seven compounds were passive diffusion because the drug concentrations in AP side were far above BL side.

Some researchers [19, 20] found that the absorption of passive-transport-based compounds has to do with its polarity. The greater the polarity of the compound is, the smaller the *P*_app_ value is. Coincidently, just from the point of view of each values, smaller *P*_app_ of TSG, EG, and PL in this research also proved this point.

TSG and EN both showed low recovery rates and low *P*_app_ in this research. We predict that large partitions of EN and TSG may accumulate or metabolise in the Caco-2 cells. TSG, EN, or their metabolites may be released from the Caco-2 cells and display their corresponding effects subsequently. However, further validation must be carried out to confirm this hypothesis.

The quantitative relationships in researches between physical-chemical properties values and *P*_app_ displayed higher correlation Gibbs energy, heat of form, and apparent permeability coefficient. The higher the Gibbs energy was, the higher the apparent permeability coefficient was. The same thing applied to the heat of form. This was in accordance with a previous research [21] showing the Gibbs (free) energy contributions to the membrane partitioning of lipidated proteins. Although the mechanism why predicted boiling point, melting point, critical temperature might affect the apparent permeability coefficient, this research provided basic clues for the further research.

## Figures and Tables

**Table:** (a) Isocratic elution procedure, detection wavelength, and *R*_*t*_ of EN, AE, RL, PL, and EG

Sample	Detection wavelength (nm)	B%	*R*_*t*_ (min)
EN	287	75	11.24
AE	254	80	5.042
RL	306	45	8.423
PL	306	40	6.568
EG	280	55	12.11

**Table:** (b) Gradient elution procedure, detection wavelength, and *R*_*t*_ of TSG and RN

Sample	Detection wavelength (nm)	B%			*R*_*t*_ (min)
		0 min	5 min	10 min	
TSG	320	40	55	70	6.513
RN	254	85	/	90	5.307

Mobile phase A: 0.1% H_3_PO_4_.

Mobile phase B: methanol.

**Table 2:** The linear equation, linear range, and correlation coefficient of nine analytes (*n* = 6).

Sample	Linear equation	Related coefficient	Linear range (*μ*mol)
TSG	*Y* = 29836*X* + 0.1657	0.9987	4.305 × 10^−6^–3.444 × 10^−4^
EN	*Y* = 18775*X* + 0.0051	1	2.664 × 10^−5^–1.066 × 10^−3^
RN	*Y* = 22458*X* + 0.2069	0.9992	2.322 × 10^−5^–9.288 × 10^−4^
AE	*Y* = 23601*X* − 0.1580	0.9996	2.664 × 10^−5^–1.066 × 10^−3^
RL	*Y* = 50841*X* + 0.2434	0.9993	1.733 × 10^−5^–3.466 × 10^−4^
PL	*Y* = 27231*X* + 0.1174	0.9999	6.892 × 10^−6^–6.892 × 10^−4^
EG	*Y* = 25907*X* − 0.0244	0.9999	7.040 × 10^−6^–2.534 × 10^−3^

**Table 3:** The apparent permeability coefficient (*P*_app_) values (Mean, *n* ≥ 3, cm/s) and recovery rates (Mean ± SD, *n* ≥ 3) of the seven analytes.

Compound	TSG	EN	RN	AE	RL	PL	EG	PR	AT
*P*_app_ value (cm/s)	2.372 × 10^−9^	2.483 × 10^−9^	1.707 × 10^−8^	1.952 × 10^−8^	1.778 × 10^−8^	4.917 × 10^−9^	2.391 × 10^−9^	6.075 × 10^−8^	1.668 × 10^−8^
Recovery rate (%)	17.39 ± 1.600	14.48 ± 2.504	96.96 ± 7.377	42.36 ± 6.323	62.39 ± 6.210	59.94 ± 9.90	33.95 ± 7.06	117.1 ± 8.85	125.2 ± 33.60

**Table 4:** The apparent permeability coefficient (*P*_app_) values (Mean, *n* ≥ 3, cm/s) and recovery rates (Mean ± SD, *n* ≥ 3) of the seven analytes.

Compound	TSG	EN	RN	AE	RL	PL	EG	PR	AT
Molecular weight	406.38	270.24	284.22	270.24	228.24	390.38	432.38	245.32	266.34
Boiling point (K)	925.79	752.86	788.86	761.48	675.11	890.96	968.71	655.04	711.80
Melting point (K)	992.60	846.01	894.89	795.11	629.96	880.88	1096.93	450.66	524.88
Critical temperature (K)	1166.96	965.47	996.03	956.41	898.50	1111.40	1238.72	833.04	887.27
Critical pressure (Bar)	40.21	52.06	47.83	48.02	50.95	33.57	34.16	24.98	24.46
Critical volume (cm^3^/mol)	1011.50	687.50	695.50	690.50	631.50	995.50	1051.5	755.50	806.50
Gibbs energy (kJ/mol)	−795.72	−357.13	−546.42	−339.33	−91.82	−641.10	−906.41	129.98	−50.00
Molar refraction index (cm^3^/mol)	100.72	71.27	71.30	71.36	66.60	99.03	103.71	72.41	73.50
Henry's law constant	26.02	18.79	19.55	19.25	14.25	22.04	26.58	10.61	16.25
Heat of form (kJ/mol)	−1309.43	−622.31	−795.27	−597.23	−273.94	−1132.12	−1480.49	−173.06	−427.56
Log *P* (by ChemDraw)	0.83	1.74	1.2	1.07	3.06	1.22	−0.1	2.33	0.50
Clog *P* (by ChemDraw)	0.6538	3.617	3.529	2.700	2.833	1.517	1.650	2.444	−0.1086
Clog *P* (by OSIRIS)	0.71	2.93	2.44	2.29	3.12	1.64	0.82	2.41	0.41
Log *S* (by OSIRIS)	−2.45	−4.19	−4.15	−4.02	−2.86	−3.21	−4.07	−3.2	−2.02

**Table 5:** Relationships between physical-chemical properties values and apparent permeability coefficient (*P*_app_).

Correlation coefficient and significance	Pearson's correlation coefficient	Significance (*p*)
Molecular weight	—	—
Boiling point	−0.687	0.041
Melting point	−0.768	0.016
Critical temperature	−0.703	0.035
Critical pressure	—	—
Critical volume	—	—
Gibbs energy	0.751	0.020
Molar refraction index	—	—
Heat of form	0.701	0.035
Log *P* (by ChemDraw)	—	—
Clog *P* (by ChemDraw)	—	—
Clog *P* (by OSIRIS)	—	—
Log *S* (by OSIRIS)	—	—

—: data were not listed when significance (*p*) was higher than 0.05.

## Abbreviations

AE: Aloe-emodin
ALP: Alkaline phosphatase
AP: Apical side
AT: Atenolol
BL: Basolateral side
Clog *P*: Calculated octanol-water partition coefficient
DMEM: Dulbecco's Modified Eagle's Medium
DMSO: Dimethyl sulfoxide
EN: Emodin
EG: Emodin-8-*O*-*β*-D-glucopyranoside
FBS: Fetal bovine serum
HEPES: N-2-Hydroxyethylpiperazine- *N*′-2- ethanesulfonic acid
HPLC: High performance liquid chromatography
Log *S*: The aqueous solubility of a compound (estimated log *S* value is a unit stripped logarithm (base 10) of the solubility measured in mol/liter)
*P*_app_: Apparent permeability coefficient
PBS: Phosphate buffer solution
PL: Polydatin
PMR: Polygonum Multiflorum Radix
PMRP: Polygoni Multiflori Radix Praeparata
PPRC: The Pharmacopoeia of the People's Republic of China
PR: Propranolol
RN: Rhein
RL: Resveratrol
*R*_*t*_: Retention time
TCM: Traditional Chinese medicine
TEER: Transepithelium electrical resistance
TSG: 2,3,5,4′-Tetrahydroxystilbene-2-*O*-*β*-D-glucoside
TG: Triglyceride
NAFLD: Nonalcoholic fatty liver disease.
